# Reactionary Medicine

**Published:** 1894-06-16

**Authors:** 


### The Hospital

An Institutional Journal of

The Medical Sciences and Hospital Administration, with

Vol. XVI.—No. 403.] AN EXTRA NURSING SUPPLEMENT. [June 16, 1894.

### Reactionary Medicine.

"Adversity," it is said, "makes us acquainted with strange bedfellows." But the self-respecting man—and he alone is worthy of serious consideration—will rather sleep under the canopy of the winter sky than associate himself with those who seek unworthy or doubtful ends. Self-preservation is one of the primary instincts of all living forms; but in creatures endowed with minds and the capacity for morals, and more especially in men who have received all the advantages of a liberal and scientific education, there are, and must be, many considerations which take precedence even of self-preservation. Medical men, we hold, should combine and stand loyally by each other for mutual preservation and just elevation. But *noblesse oblige*; in all their combinations they must remember that they are men of education, that they have a reputation for intellectual capacity as well as for honourable conduct to maintain, and that whatever objects they seek must be such, and such only, as are consistent with their position in the State as healers and promoters of the physical well-being of all Her Majesty's subjects.

From this point of view every medical man who has noted the under-current of feeling which has for some time been running against any recognition whatever of midwives as a class, and any attempt whatever to improve their capacity for doing their work well, must have felt that certain members of his profession were acting the part, not of trade unionists merely, but of trade unionists of the most limited vision and of the feeblest possible motive power. For of every variety of motive power which makes appeal to the public conscience and the public mind that of naked self-interest is the feeblest and the least likely to prevail.

The Association which now calls itself by the name of the "Incorporated Medical Practitioners Association," but for which the only appropriate name is surely the medical "Cave of Adullam," since every discontented spirit which "frets and fumes its brief hour" on the stage of medicine, appears to have rushed into it—that Association has boldly reared its flag in defiance of medical progress and in favour of a reactionary trade-unionism of the most money-loving and least exalted type. In the forefront of a new programme issued by the Association there is announced a determined resistance to the Midwives' Registration Bill. Credit is taken for "the important part played in postponing the passing of the Bill," and for "opposing by all means in their power this legislative step."

But now, let this question of midwives' registration be looked at from the standpoint of high medical policy, from the standpoint of manly and dignified general practice, and from the still more important standpoints of the humane instincts of the community at large and of the sorrows and sufferings of the poorest class of women who are face to face with childbirth. There is a General Medical Council, as we all know, and though we are of opinion that that Council has taken too limited a view of its own powers and duties, yet we cannot but feel that it is a Council of the most capable men in medicine, and has the best interests of the public and the profession at heart. Let us for a single moment pause here and ask ourselves what the General Medical Council would be if it consisted of such persons and put forth such a programme as the council of the Incorporated Medical Practitioners consists of and has put forth? It would be a council devoting itself to the sale of medical practices and the pocketing of small commissions thereupon, to the opposing of practical medical progress in its application to the most necessitous classes of the people, to the acting as an agency for fire insurance offices, and other like pursuits of a pettifogging commercial character. Would such a council in any sense represent the science and the art, the public distinction and the social elevation of the medical profession of the present day? It would degrade medicine and drag it down to the level of a council of commission agents dabbling in every conceivable commercial speculation by which a miserable and non-professional penny could be turned.

But the General Medical Council, in contrast with such a council as reactionary practitioners seem to prefer, is a dignified and cultured body, endowed with very considerable capacity for broad and reasonable medical statesmanship. What does this leading medical organisation pronounce upon the question of midwives' registration? At its recent sittings it resolved that "This Council regards the absence of public provision for the education and supervision of midwives as productive of a large amount of grave suffering and fatal disease among the poorer classes, and urges upon the Government the importance of passing into law some measure for the education and registration of midwives." That is the high policy which those recognized leaders of the profession have definitely decided to pursue on the "Midwives' Registration" question. The profession now knows which way its leaders are moving. Those who agree with the policy of the Council should promptly declare themselves. Those who disagree have anticipated the Council and proclaimed their disagreement.

Now let the question be looked at from the standpoint of dignified general practice. Here also we have a set of concrete facts which show us the trend of mind of those practitioners who take what we will venture to call the public spirited and statesmanlike view of the question. Some seventy two members of the Lancashire and Cheshire Branch of the British Medical Association have thought it their duty to enter a public protest against those medical reactionaries in the branch who sought to commit their medical brethren to an attitude of antagonism against the education and registration of midwives. They dissent from the reactionaries on the altogether honourable and humane ground that "it is against the best traditions of the medical profession to oppose any reasonable measure which has for its object the protection of the lives and health of the public." Here we have self-respecting and dignified general practice taking up a position worthy of a profession which is at once cultured and humane; a position which all thoughtful persons in all ranks of the community, from the humblest labourer's wife, who cannot pay a doctor's fee in her confinement, to the highest statesman in the land, can appreciate and generously approve.

Argument from the point of view of common humanity and the sufferings and premature deaths of large numbers of poor mothers at the hands of unskilled midwives is quite superfluous, and will certainly not be attempted here. What does really surprise us is the line of reasoning followed by those who oppose registration. They tell us of the mischief which is done by the granting of certificates by private practitioners and unauthorised organisations, certificates which are fraudulently used as diplomas by those who practice midwifery; and of the many untimely deaths which are caused by want of skill on the part of the present class of midwives. Quite so! But can they not see that any argument of this kind is an argument for, instead of against, the proper education and registration of midwives? If education and registration are made compulsory, then it will be the Medical Council which will prepare a scheme of education, which also will prescribe the indispensable conditions of registration, and which, moreover, will supervise and control qualified midwives from the beginning to the end of their professional career. From the very date that registration is determined upon, from the very moment, the granting of certificates by private medical men or unauthorised organisations, will cease. To oppose registration on the ground of these unauthorised certificates is the same as asserting that a horse will gallop better when his legs are cut off. The truth is that there is no honest ground but one on which registration is opposed. That ground is that the opponents fear an injury to their professional emoluments. We sympathise with the fear; and if that position were consistently taken up we should at least respect the arguments of the opponents, although we might disagree with them. But we do not think for a moment that the struggling practitioner would be the poorer if midwives were educated and registered. On the contrary, we hold that registration would reduce the number of practising midwives very greatly, and would, moreover, both raise the status of the general practitioner and put money into his pocket.

